# Metasecretome-selective phage display approach for mining the functional potential of a rumen microbial community

**DOI:** 10.1186/1471-2164-15-356

**Published:** 2014-05-12

**Authors:** Milica Ciric, Christina D Moon, Sinead C Leahy, Christopher J Creevey, Eric Altermann, Graeme T Attwood, Jasna Rakonjac, Dragana Gagic

**Affiliations:** Animal Nutrition and Health, AgResearch Ltd, Palmerston North, 4442 New Zealand; Institute of Fundamental Sciences, Massey University, Palmerston North, 4442 New Zealand; Institute of Biological, Environmental & Rural Sciences, Aberystwyth University, Penglais, Aberystwyth, Ceredigion, SY23 3DA UK

**Keywords:** Phage display, Nxt generation sequencing, Metagenomics, Rumen, Cellulosome, Surface and secreted proteins

## Abstract

**Background:**

*In silico*, secretome proteins can be predicted from completely sequenced genomes using various available algorithms that identify membrane-targeting sequences. For metasecretome (collection of surface, secreted and transmembrane proteins from environmental microbial communities) this approach is impractical, considering that the metasecretome open reading frames (ORFs) comprise only 10% to 30% of total metagenome, and are poorly represented in the dataset due to overall low coverage of metagenomic gene pool, even in large-scale projects.

**Results:**

By combining secretome-selective phage display and next-generation sequencing, we focused the sequence analysis of complex rumen microbial community on the metasecretome component of the metagenome. This approach achieved high enrichment (29 fold) of secreted fibrolytic enzymes from the plant-adherent microbial community of the bovine rumen. In particular, we identified hundreds of heretofore rare modules belonging to cellulosomes, cell-surface complexes specialised for recognition and degradation of the plant fibre.

**Conclusions:**

As a method, metasecretome phage display combined with next-generation sequencing has a power to sample the diversity of low-abundance surface and secreted proteins that would otherwise require exceptionally large metagenomic sequencing projects. As a resource, metasecretome display library backed by the dataset obtained by next-generation sequencing is ready for i) affinity selection by standard phage display methodology and ii) easy purification of displayed proteins as part of the virion for individual functional analysis.

**Electronic supplementary material:**

The online version of this article (doi:10.1186/1471-2164-15-356) contains supplementary material, which is available to authorized users.

## Background

Microorganisms account for a major proportion of our planet’s biological diversity and thus present an enormous and largely unknown resource that can be utilised in the discovery of novel genes, bioactive molecules [1] and new biocatalysts. These may be exploited to improve industrially relevant processes [2]. The traditional approach to tap into this resource is via the cultivation of microorganisms and screening for individual strains with the desired phenotype(s). However, more than 90% of microbes in complex microbial communities are not culturable by standard laboratory techniques [3]. The nature of these complex microbial communities is being realised in culture-independent approaches, collectively known as metagenomics [4]. These approaches range from the amplification and deep sequencing of phylogenetically informative genes and regions within community DNA (such as the 16S rRNA gene) to assess community structure, shotgun sequencing of community DNA to determine their coding potential, through to targeted functional screens of libraries constructed from community DNA [5–7].

The fermentative forestomach of ruminant animals, known as the reticulo-rumen, is one of the most complex microbial ecosystems investigated via metagenomic studies [8]. Since the 1980s, the rumen has been used as a source for the discovery of enzymatic activities involved in the degradation of the lignocellulosic components of the plant cell wall for both agricultural and biofuel production applications [9–11]. It is estimated that the rumen harbours up to 3,000 bacterial species, the majority belonging to the phyla Firmicutes and Bacteroidetes, with species belonging to the Proteobacteria, Fibrobacteres and Spirochaetes also present [12–15].

Rumen microorganisms metabolise plant structural carbohydrates using a broad spectrum of *C*arbohydrate-*A*ctive en*Zymes*, commonly known as CAZymes [16, 17], including glycoside hydrolases (GHs), carbohydrate esterases (CEs), glycosyltransferases (GTs) and polysaccharide lyases (PLs). Many CAZymes are modular, containing one or more catalytic domain(s) and ancillary non-catalytic modules including carbohydrate binding modules (CBMs). CBMs are thought to increase the efficiency and specificity of the catalytic module by attachment to a specific sugar moiety [18–20]. A feature of some rumen microbes is the association of CAZymes with cell wall-bound multienzyme structures called cellulosomes [21, 22]. Cellulosomal CAZymes contain signature domains (dockerins) that anchor the enzymes to cognate domains (cohesins), of a bacterial envelope-bound scaffold composed of one or more proteins called scaffoldins [23]. The synergistic action of CAZymes that assemble as cellulosomes is usually associated with improved fibrolytic function, rendering these surface complexes a desirable target for identification and functional characterisation [24, 25].

Secreted CAZymes, including the non-catalytic cellulosome components (e.g. scaffoldins), are but a small fraction of the surface and secreted proteins that make up the “secretome” of a microbial community (metasecretome) [26–29]. Proteomics, despite its power in analysing water-soluble proteins, allows a very limited detection of cell-surface and membrane proteins. Furthermore, at the scale of microbial communities, proteomic approaches are highly dependent on the preparation method and only detect the most abundant secreted or membrane proteins, with the low-abundant proteins escaping identification [30, 31]. Most secretome proteins have membrane-targeting signal sequences and transmembrane α-helices, including the classical Type I, Type II lipoprotein, Type IV prepillin and the twin arginine translocon (Tat) signal sequences [32]. These sequences can be used to predict secretome proteins from sequenced genomes using various algorithms (e.g. SignalP [33], SecretomeP [34], TMHMM [35], and PRED-LIPO [36]). Despite the ability to predict metasecretome proteins *in silico*, direct analysis of metasecretome proteins (whose coding sequences are predicted to comprise 10 – 30% of total ORFs within the metagenome) is desirable to confirm their functions [14, 37–39].

Recently, phage display technology has been adapted for the direct selection and display of secretome proteins, and was applied at a single genome scale to *Lactobacillus rhamnosus* and *Mycobacterium tuberculosis*[40, 41]. Sequence analysis and affinity screenings of the resulting phage display secretome libraries allowed characterisation of surface proteins with functions of interest [40–43]. This technology has potential application at a scale of an entire microbial community, where cultivation-independent methods are required to enable discovery and functional characterisation of products encoded by complex microbial communities. Phage display allows affinity screening of large libraries for functions of interest due to the physical connection of the displayed proteins to the phage-encapsidated coding nucleic acid; displayed proteins can also be easily purified as part of the virion [44–46]. However, given that the published secretome-selective phage display system is limited by the *E. coli* inner membrane translocation systems for the display of secretome proteins, it was uncertain whether this method would limit the diversity of displayed secretome proteins from the taxonomically diverse species that constitute the rumen microbial community.

In this study we applied the secretome-selective phage display method at a metagenomic scale, in combination with next-generation sequencing, and showed that it efficiently displayed functionally and taxonomically diverse secretome proteins, further focusing sequencing effort onto a subset of biologically relevant sequences from a very complex microbial community. In doing so, this approach permitted the discovery of a large assortment of new secreted CAZymes from the bovine rumen microbial community, in particular, expanding the known diversity of cellulosome components, likely to be involved in ruminal fibre degradation.

## Results

### Efficiency of metasecretome phage display library selection, secretion signals and phylogenetic diversity

**Figure 1:**
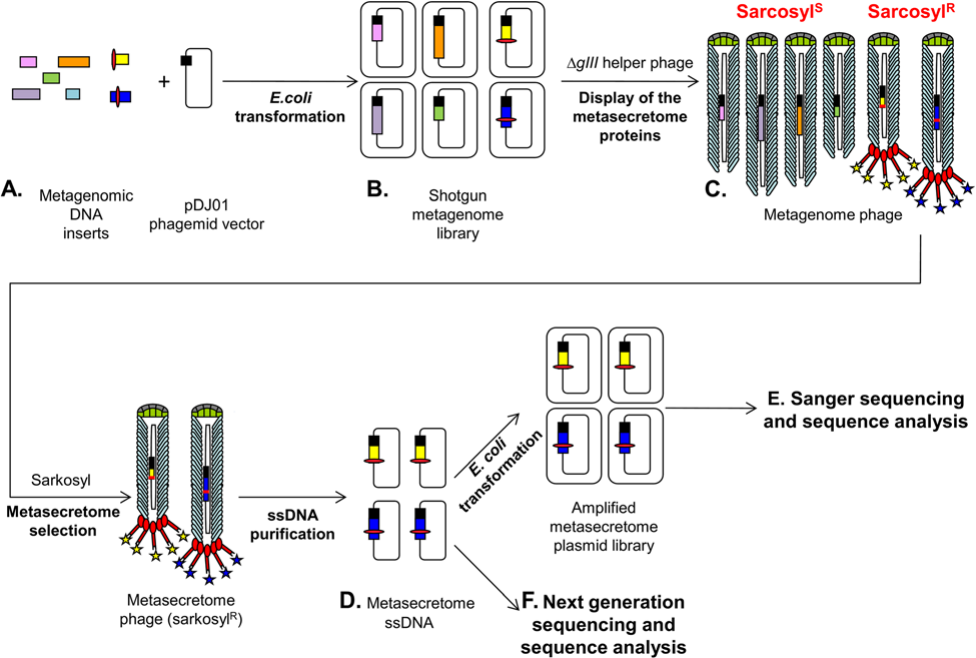
**Overview of metasecretome library construction and selection. (A)** A shotgun metagenomic library was constructed by cloning metagenomic DNA into the pIII cloning cassette of pDJ01 phagemid vector that does not contain a signal sequence. A small proportion of metagenomic inserts contain signal sequences or other membrane-targeting sequence motifs (red oval shape). **(B)** Recombinant phagemids replicate as plasmids inside the cells, or alternatively, in the presence of the helper phage, they are packaged as recombinant virions called phagemid particles (PPs). **(C)** After infection of the library with the *gIII*-deleted helper phage VCSM13d3, the PPs derived from the recombinant clones that do not contain a membrane-targeting sequence lack the pIII-made cap structure (bottom end of the metagenome phage in the figure). In contrast, the PPs derived from the recombinant phagemids that encode a membrane-targeting sequence in frame with pIII contain the cap structure formed by insert-pIII fusion. Due to the lack of the pIII virion cap, the PPs that do not encode membrane-targeting signals were disassembled in the presence of ionic detergent sarcosyl (Sarcosyl^S^), while the secretome protein-displaying PPs were resistant to sarcosyl (Sarcosyl^R^), and this was used as a basis for selection. **(D)** After the removal of ssDNA released from the disassembled Sarcosyl^S^ PPs, the ssDNA from the intact Sarcosyl^R^ PPs was purified and used to: **(E)** transform *E. coli* to obtain an amplified metasecretome plasmid library for preliminary assessment of metasecretome diversity by Sanger sequencing of clone inserts and **(F)** as a template for metasecretome analysis by next-generation sequencing.

A shot-gun library was constructed in a phagemid/helper phage secretome-selective phage system as described in Jankovic et al. [40] (see Figure 1 for schematic overview of library construction). To maximise the probability of identifying extracellular proteins involved in fibre degradation, a plant-adherent fraction of the rumen microbial community from pasture-fed cows was used as a source of DNA for library construction. A small pilot library was initially constructed in the secretome-selection phagemid vector pDJ01 [40]. The primary size of this library (before secretome selection) was 4 × 10^5^ clones, and the insert size range was approximately 0.7 to 5 kb. The library was subjected to secretome selection, producing a recombinant clone pool enriched for secretome proteins, in the form of recombinant phagemid single stranded DNA (ssDNA) [40]. To assess the efficiency of selection, ssDNA was transformed into *E. coli* TG1 and 90 individual transformants were analysed by sequencing the phagemid inserts.

**Figure 2:**
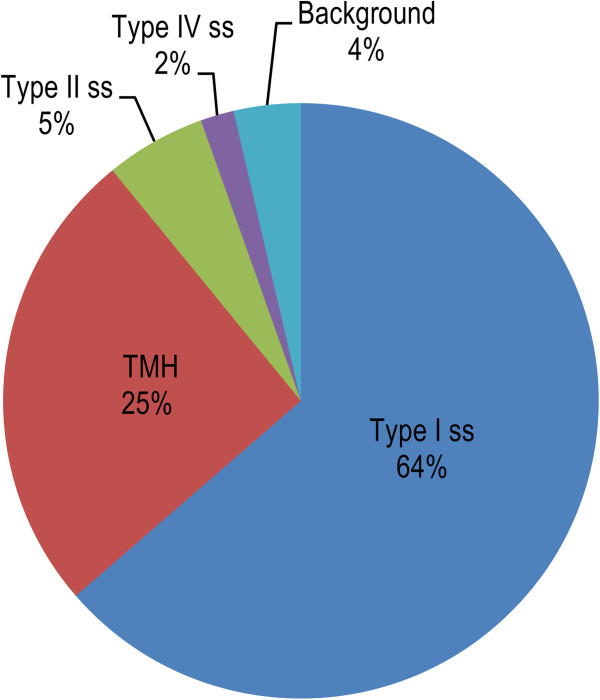
**Types of membrane-targeting signals detected in metasecretome pilot library ORFs.** Abbreviations used for membrane-targeting signal types: ss, signal sequence; Type I ss, classical ss; Type II ss, lipoprotein ss; Type IV ss, pilin-like ss; TMH, N-terminal or internal transmembrane α helix/helices; background - ORFs without membrane-targeting signal or shorter than 24 amino acids.

It was found that 85 of the 90 inserts analysed (94.4%) contained 53 distinct ORFs encoding secretome proteins with typical signal sequences in-frame with pIII. Of the remaining five inserts (5.6%), one contained an ORF encoding a polypeptide in frame with pIII that was shorter than 24 amino acid residues and was considered “background” (Figure 2). The remaining four inserts contained a single ORF without typical membrane-targeting sequence. Further analysis using SecretomeP 2.0, which discriminates between non-classically secreted proteins and cellular proteins based on amino acid composition, secondary structure and disordered regions, gave score < 0.5, which indicates that polypeptide encoded by this ORF is not secreted via non-classical secretion pathways. BLAST analysis was used to predict localisation of the putative protein based on sequence homology. The protein showed homology to a conserved hypothetical protein with predicted cytoplasmic localisation, and was therefore also considered “background” that was not eliminated by selection (Figure 2).

Based on the average proportion of secretome ORFs in bacterial genomes (~20%), and the probability of the insert being in the same orientation (50%), and in-frame (33.3%) with gene *gIII* to create an in-frame protein fusion with pIII, we expect only ~3.3% of the inserts in the library to be selected. Therefore, the efficiency of selection was estimated by comparing the frequency of secretome insert-containing recombinant phagemids after selection 85/90 (94.4%) with the theoretically predicted frequency (3.3%). The enrichment of the secretome insert-containing recombinant library clones was 29-fold, indicating that the stringency of selection was high, and that most recombinant phagemids containing non-secretome inserts (background) were eliminated.

The types of membrane-targeting signals predicted from the pilot metasecretome phage display library ORFs are summarised in Figure 2, while the membrane-targeting sequences and detailed analysis are presented in Additional file 1. The majority of ORFs (35) contained type I signal sequences while the remainder consisted of transmembrane α-helices with N-terminal transmembrane anchors (8), multiple transmembrane α-helices or single internal transmembrane α-helices (6), type II or lipoprotein signal sequences (predicted in three ORFs), and a single type IV (pillin-like) signal sequence. Selection of protein-pIII fusions containing type II signal sequences or transmembrane helices has been observed in genomic secretome-selective display [40], despite the fact that the native pIII signal sequence is type I. It appears that a predicted transmembrane α-helix and dependence on the SecYEG translocon is the condition for assembly of sarcosyl-resistant recombinant virions. The absence of the Tat signal sequences likely stems from the fact that their export depends on the specific TatABC translocon, involved in the transport of folded substrates. It was shown that Tat pathway is not suitable for targeting of the pIII fusions to the virion, since protein-pIII fusion typically folds in the oxidising environment of the *E. coli* periplasm, in contrast to the Tat-dependent proteins that fold in the reducing environment of the cytoplasm [47, 48].

To identify the organisms from which metasecretome clones were derived, taxonomic assignments were designated for the predicted proteins of each insert, based on the best BLASTX hits, where the E-value was less than 1 × 10^−5^ and query coverage greater than 30%. The most abundant assignments were to the genera *Prevotella* (13%), *Clostridium* (10%), *Butyrivibrio* (7%), *Ruminococcus* (6%), *Bacteroides* (6%) and *Fibrobacter* (4%); genus-level assignments could not be made for 50% of the inserts analysed. These results indicate that the metasecretome selection method captured representatives of the main genera comprising the core bovine rumen microbiome, as previously determined by pyrosequencing of 16S rRNA genes of other rumen microbial communities [15, 49].

### Metasecretome characterisation by next-generation sequencing

The small scale of the pilot metagenome library and metasecretome selection that included transformation bottleneck and standard Sanger sequencing did not allow access to the large diversity of the rumen microbial metasecretome. Therefore, to improve on the representation of the metasecretome, an upscaled primary metagenomic library was constructed with a final size (before selection) of ~5 × 10^6^ primary clones. Furthermore, the secretome selection protocol was combined with the next-generation sequencing of inserts. After secretome selection [40], the inserts from the resulting metasecretome ssDNA pool were PCR-amplified and processed by enzymatic and mechanical shearing to fragments of a suitable size range (600 - 800 bp) for 454 GS FLX sequencing. A total of 691,206 obtained sequence reads were obtained and processed (including trimming, low complexity filtering and de-replication), resulting in 153,002 de-replicated reads that were further analysed (see Additional file 2 for the NGS summary and statistics).

**Figure 3:**
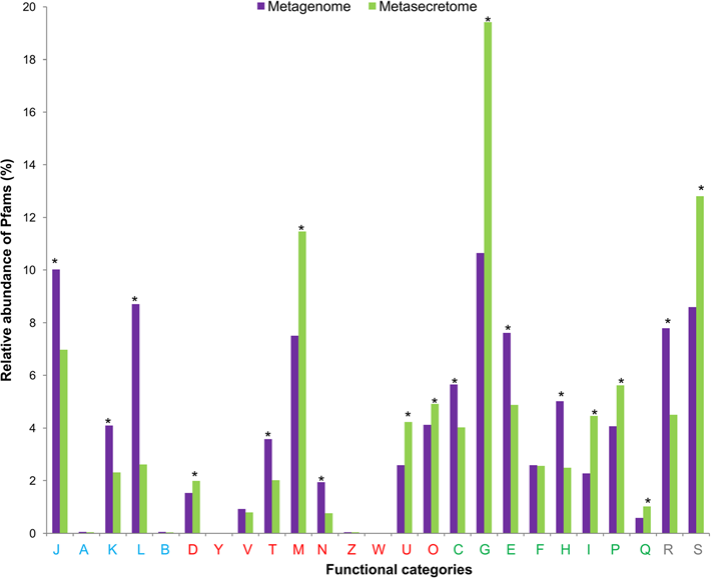
**Relative abundances of Pfams within the metagenome and metasecretome-enriched sequence datasets.** Relative abundances of IMG/M annotated COG-based functional categories of protein family (Pfam) conserved domains within the metagenome (purple bars) and metasecretome-enriched (green bars) sequence datasets. Abbreviations for the functional categories, grouped by general functional role: **Information storage and processing (blue font)**: J – Translation, ribosomal structure and biogenesis, A – RNA processing and modification, K – Transcription, L – Replication, recombination and repair, B – Chromatin structure and dynamics; **Cellular processes and signalling (red font):** D – Cell cycle control, cell division, chromosome partitioning, Y – Nuclear structure, V – Defence mechanisms, T – Signal transduction mechanisms, M – Cell wall/membrane/envelope biogenesis, N – Cell motility, Z – Cytoskeleton, W – Extracellular structures, U – Intracellular trafficking, secretion and vesicular transport, O – Posttranslational modification, protein turnover, chaperones; **Metabolism (green font):** C – Energy production and conversion, G – Carbohydrate transport and metabolism, E – Amino acid transport and metabolism, F – Nucleotide transport and metabolism, H – Coenzyme transport and metabolism, I – Lipid transport and metabolism, P – Inorganic ion transport and metabolism, Q – Secondary metabolites biosynthesis, transport and catabolism; **Poorly characterized (grey font):** R – General function prediction only, S – Function unknown. Significant difference between metasecretome and metagenome datasets within given functional category is represented by asterisks (* *P* ≤ 0.001).

To predict the putative functions that were enriched in the metasecretome library, the metasecretome sequence data was compared to a 454 GS FLX shotgun sequenced metagenome derived from the plant-adherent rumen microbial fraction of two New Zealand cows grazing a similar pasture-based diet (data not published). Annotation of the metasecretome and metagenome sequence reads via IMG/M system [50] resulted in 35% and 49% Pfam [51] assignments of the total protein coding genes, respectively, which were further categorised into COG-based functional categories (Figure 3). The functional category with the most assignments was “carbohydrate transport and metabolism” for both the metagenome (10.6%) and the metasecretome datasets (19.4%) (Figure 3, bar G). Metasecretome phage display also enabled enrichment of proteins predicted to be involved in the “cell wall/membrane/envelope biogenesis” (Figure 3, bar M) and peptides with unknown function (Figure 3, bar S). Proteins of unknown function are generally overrepresented in the secretome fraction of bacterial genomes [52, 53], and their enrichment is consistent with enrichment of the metasecretome. In contrast, the functional categories of “replication, recombination and repair” (Figure 3, bar L) and “coenzyme transport and metabolism” (Figure 3, bar H), comprised mainly of intracellular proteins, were under-represented in the metasecretome dataset.

### Carbohydrate-active enzyme (CAZyme) diversity and abundance of cellulosome components within the metasecretome selected library

The metasecretome (and metagenome) ORFs were analysed using the dbCAN database to determine the diversity of CAZyme families captured by the metasecretome selection (Table 1). The dbCAN database uses Hidden Markov Models (HMMs) of the signature domain regions for all CAZyme families, and incorporates the most complete set of metagenomic CAZyme genes published so far [54]. The analysis identified 12,565 putative CAZyme hits in the metasecretome library with a significant match to at least one catalytic domain or associated module belonging to 196 different CAZy families while the analysis of metagenome (21,823 hits) identified 318 CAZy families (Additional file 3).

**Table 1:** **Comparison of CAZyme classes between plant-adherent rumen microbial metasecretome and metagenome datasets**

CAZyme class	Count MS	Distribution MS	Count MG	Distribution MG
*Carbohydrate-binding modules*	1038	8.3%	cpo1656	7.6%
*Carbohydrate esterases*	1499	11.9%	2235	10.2%
*Glycoside hydrolases*	7639	60.8%	11606	53.2%
*Glycosyl transferases*	793	6.3%	5126	23.5%
*Polysaccharide lyases*	382	3.0%	524	2.4%
*Auxiliary activities*	67	0.5%	451	2.1%
Cellulosome components*	1147 (577)	9.2% (7.2%)	225 (207)	1.0% (0.9%)
SLH	46 (34)	0.37% (0.43%)	77 (72)	0.35% (0.33%)
cohesins	52 (44)	0.41% (0.55%)	27 (27)	0.12% (0.12%)
dockerins	1049 (499)	8.35% (6.25%)	121 (108)	0.55% (0.50%)
Total*	12565 (7978)	100.0%	21823 (21607)	100.0%

Abbreviations: MS, metasecretome dataset; MG, metagenome dataset. *Numbers in parentheses refer to the CAZYme hits clustered at 100% sequence identity to remove duplicity and were used in analysis of cellulosome hit frequencies.

In both datasets we captured an assortment of cellulases, endoxylanases, carbohydrate debranching enzymes and oligosaccharide-degrading enzymes, as well as a suite of carbohydrate esterases responsible for deacetylation of xylans and xylo-oligosaccharides, and polysaccharide lyases. The GH profile of the metasecretome dataset was also similar to other reported bovine metagenomes except that GH53 (exclusive β-1,4-galactanase), responsible for degradation of galactans and arabinogalactans, and GH43 (various oligosaccharide degrading enzymes) were detected in abundance [13, 14]. When compared to the control metagenome dataset, xyloglucanases GH16 and GH74, and other oligosaccharide degrading enzymes belonging to GH2 and GH3 families occurred at higher frequency in the metasecretome dataset. In contrast, endohemicellulases (GH8, GH10) and debranching enzymes (GH51, GH67, GH78) occurred at lower frequency in the metasecretome dataset. Other GH class members that were enriched and significantly more abundant in the metasecretome compared to the metagenome dataset belong to families GH124 (cellulosomal endoglucanases; 14.3-fold enrichment), GH55 (β-1,3-glucanases; 6.5-fold) and GH92 (α-mannosidases; 5.9-fold). In the CAZy database, GH family 124 has only one characterised enzyme while a prokaryotic representative of GH family 55 has not been yet characterised. The CBMs prevalent in metasecretome, CBM67 and CBM40, are usually associated with catalytic modules of GH78 and GH33; however, representatives of these GH families were not found in large numbers in this dataset. In concordance with their extracellular function, several CE families involved in hemicellulose (CE1, CE3, CE7) and pectin (CE8) degradation detected in metasecretome were enriched and significantly more abundant than in the metagenome. The analysis of glycosyl transferases (GTs), the enzymes that assemble glycans (glycoproteins, glycolipids, oligosaccharides), showed a decrease from 23.5% in the metagenome to 6.3% in the metasecretome, consistent with the evidence that the majority of bacterial GTs are located in the cytoplasm [55].

**Figure 4:**
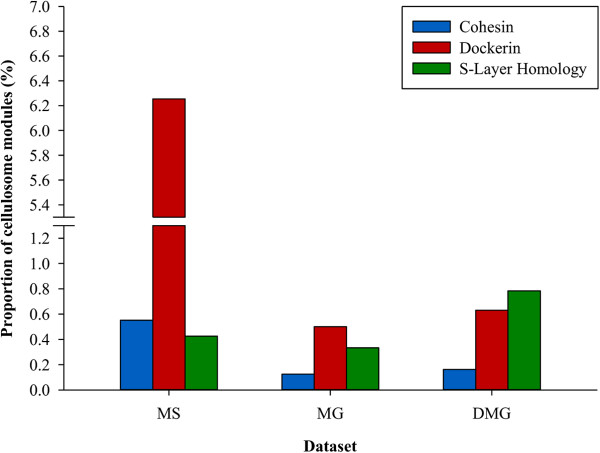
**Frequency of cellulosome modules in three bovine rumen microbial datasets.** Frequency of three cellulosome signature modules: cohesin (blue); dockerin (red) and surface layer homology (SLH) domains (green) were compared between three datasets: MS, metasecretome; MG, metagenome (both derived from the plant-adherent rumen microbial community fraction isolated from fistulated pasture-grazing dairy cows) and DMG, published deep-sequenced metagenome dataset derived from the bovine switchgrass-adherent microbiome, isolated from switchgrass that was incubated in the rumen of a fistulated cow for 72 h [14]. The total number of distinct CAZyme hits, obtained after clustering all dbCAN hits at 100% sequence identity threshold using the CD-HIT algorithm [76], were: MS, 7,978; MG, 21,607; DMG, 123,223.

A high number of putative components [cohesins, dockerins and surface layer homology (SLH) modules] of complex carbohydrate-degrading surface complexes – cellulosomes were detected (Figure 4). Analysis of metasecretome ORFs with hits to cellulosome-associated modules, clustered at 100% sequence identity to remove duplicity, revealed that 6.3% of the total clustered CAZyme hits were to dockerins (Table 1). Of those, 4.5% hits were to a HMM representing a single dockerin repeat; 1.7% were to presumably complete dockerin domains (containing two hits to dockerin repeat HMMs) and 0.1% were to single dockerin repeat in combination with another CAZyme module. Two other modules present in cellulosomes, cohesin and SLH, were also detected (0.6% and 0.4%, respectively).

**Figure 5:**
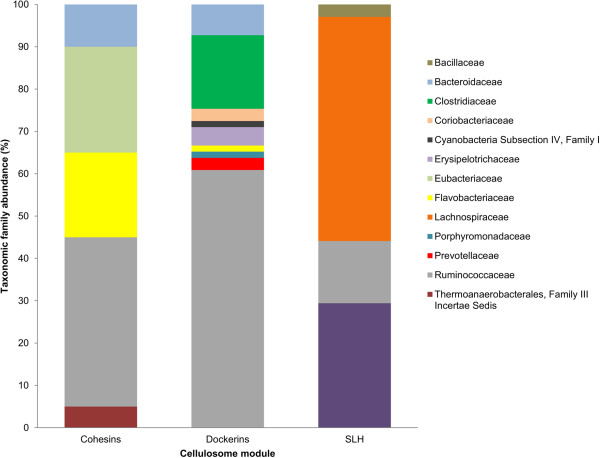
**Phylogenetic diversity of cellulosome modules predicted in the rumen metasecretome-enriched dataset.** Translated metasecretome ORFs that were predicted to contain cellulosome modules (cohesin, dockerin and SLH domains) were compared to the non-redundant protein database using BLASTP. Family-level taxonomic assignments were made for the host organism of the best BLAST hit and the chart shows the abundance of each family for each cellulosome module. For the dockerin data, only sequences that contained two dockerin modules (N = 69) are shown.

The phylogenetic diversity of the translated CAZyme ORFs predicted to contain cellulosome modules was determined by family-level taxonomic assignment based on the best BLASTP hit (Figure 5), and the recently proposed reclassification of *Clostridium* spp. based on extensive molecular phylogenetic data [56, 57]. Around two thirds of cohesin modules containing sequences were assigned to the Firmicutes [including Ruminococcaceae (40%) and Eubacteriaceae (25%)], with the remaining assigned to Bacteroidetes [Flavobacteriaceae (20%) and Bacteroidaceae (10%)]. The vast majority of dockerin-containing sequences were assigned to the Firmicutes [including Ruminococcaceae (61%) and Clostridiaceae (17%)] and Bacteroidetes representation was mainly within the Bacteroidaceae (7.3%), and Prevotellaceae (2.9%). Among the best BLASTP hits, many were to species that have been previously reported as cellulosome-producers, such as *Acetivibrio cellulolyticus*, *Clostridium acetobutylicum*, *Ruminococcus albus*, *R. flavefaciens*, *Ruminiclostridium cellulolyticum* (formerly *Clostridium cellulolyticum*), *Ru. josui* (formerly *C. josui*) and *Ru. thermocellum* (formerly *C. thermocellum*) [22]. In contrast, 97% of putative SLH domains were assigned to Firmicutes (including 53% to Lachnospiraceae, 29% to Veillonellaceae and 15% to Ruminococcaceae).

### Phylogenetic diversity of the selected metasecretome

**Figure 6:**
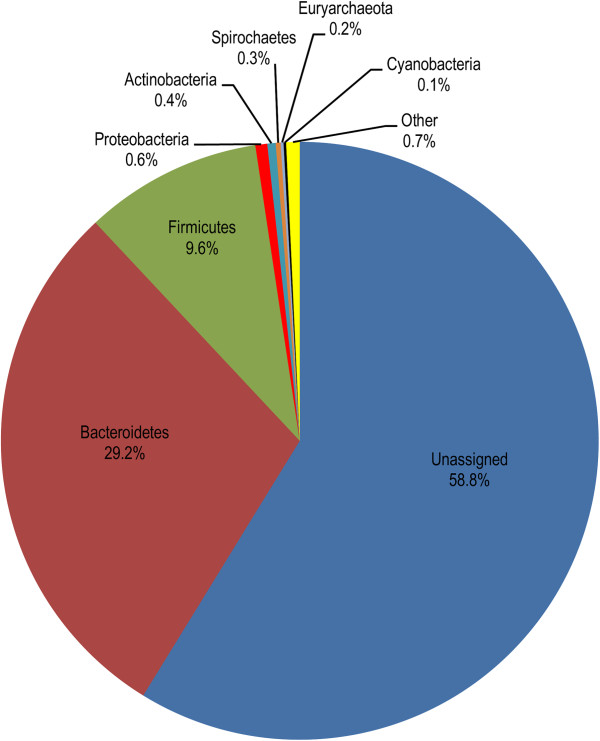
**Phylogenetic profile of the metasecretome-enriched dataset.** The taxonomic assignment of the metasecretome reads derived from the rumen adherent microbial fraction was based on distribution of best BLAST hits of protein-coding genes at 30% BLAST identity. Slices of a pie chart are corresponding to the percentage of total best BLAST hits at phyla level. The “Other” contains ORFs with database hits belonging to a phylogenetic group of low abundance in the dataset (<0.1%), while the “Unassigned” corresponds to predicted ORFs with hits below 30% identity cut-off.

We used an IMG/M similarity-based binning approach for the taxonomic assignment of the predicted protein-coding sequences, and to determine their phylogenetic distribution (Figure 6). The majority of assigned sequences belong to Bacteria (40.9%), 0.2% to Archaea and 0.1% to Eukaryota, while 58.8% remained unassigned. Approximately 28% of the sequences assigned to Eukaryota were most similar to fungi and around 14% to plants, which may reflect the presence of low levels of plant and fungal material within our plant-adherent microbiome samples. Virus hits were rare (0.004%). At the phylum level, Bacteroidetes (29%) and Firmicutes (10%) dominated, with minor contributions from Proteobacteria, Actinobacteria, Spirochaetes and Cyanobacteria. The main taxonomic assignments are in agreement with predominant phyla determined in the 16S rRNA gene based studies of bacterial diversity of other rumen microbial communities [15]. A higher representation of sequences from Gram-negative bacteria was apparent in the metasecretome dataset relative to the metagenome dataset. This was consistent with taxonomic representation of the metasecretome pilot library inserts, and might be due to a somewhat higher efficiency of Gram-negative relative to Gram-positive membrane-targeting signals in *E. coli* as a host strain.

## Discussion

Improving the digestive processes of ruminant animals, or degradation of lignocellulosic feedstocks for biofuel production, requires an understanding of the enzymatic processes involved in the depolymerisation of plant structural carbohydrates. The majority of the information currently available has been generated from the study of individual microbes and their enzyme complements, but in nature the breakdown of plant polysaccharides is initiated by microbial consortia and their secreted enzymes. This is much more complex and difficult to study, but the recent development of high-throughput sequencing and associated metagenomic techniques opens up new opportunities to begin to understand this complex process. In this study we have assessed the rumen metasecretome, using a secretome-selective phage display technology that enables the focusing of next-generation sequence analysis to this portion of the metagenome. This is, to our knowledge, the first report of selective sequence analysis as a method to focus on the sequences encoding secreted proteins from a metagenome. The rumen microbial metasecretome is specialised for the initial degradation of plant fibre through the action of surface-associated and secreted enzymes. Consistent with this, the metasecretome display approach has considerably enriched for secretome proteins in the “carbohydrate transport and metabolism” functional category. This functional category was represented in the metasecretome dataset with a wide diversity of GH catalytic modules, assigned to 85 GH families, accompanied by a variety of CBMs (belonging to 38 CBM families), CEs (13 families) and PLs (10 families).

The selectivity of the method was apparent when the abundance of two subcategories of CAZymes: GTs and cellulosomal modules (specifically, cohesins and dockerins) were compared to corresponding groups in a metagenome dataset. The relatively lower representation of GTs in the metasecretome is consistent with the current knowledge of GTs cytosolic localisation in bacteria [58]. On the other hand, proteins containing cohesin and dockerin domains are secreted or membrane-bound, as described for several anaerobic bacteria, notably *Ru. thermocellum* and *C. cellulovorans,* and *R. flavefaciens* FD1 [58–60]. A striking difference in comparison with reports from previous rumen microbiome studies and our metagenome lies in the presence of a high frequency of putative cohesin and dockerin modules. For example, comparison of the abundance of cellulosome-associated modules in our metasecretome dataset, with those in a switchgrass-adherent bovine rumen microbial metagenomic sequence dataset [14], predicted using the same database and search parameters [54], showed a prominent enrichment for cohesin and dockerin modules (Figure 4). Other published rumen metagenomic datasets have detected even lower proportions of cellulosomal modules [13, 61, 62]. The majority of the metasecretome inserts predicted to encode dockerin and cohesin modules showed strong homology to sequences from members of the Ruminococcaceae [56]. This finding is reasonably consistent with the taxonomic affiliations of known cultivated cellulosome producing-bacteria, which are also predominantly from the Ruminococcaceae [22]. Our results suggest that, within the plant-adherent rumen microbial fraction, members of the Ruminococcaceae also have the greatest potential to produce cellulosome-like structures. A number of cohesin (10%) and dockerin (7.25%) containing inserts were assigned to the Bacteroidaceae, suggesting potential for this family to produce cellulosomes. However, currently there are no reports of cellulosome-producing organisms from this family. Interestingly, one of the earliest reported cellulosome producers, *Bacteroides cellulosolvens*[63], is now recognised as a member of the Ruminococcaceae where it has been reclassified as *Ruminiclostridium cellulosolvens*[56]. In the metasecretome dataset, almost 18% of the dockerin-encoding inserts were most similar to sequences from members of the Clostridiaceae, although curiously, cohesin-containing ORFs that are also associated with this family were not detected. In total, only 44 sequences with hits to cohesin domains were detected in this study, as compared to more than 400 predicted dockerin-containing sequences. Within the genomes of cellulosome-producing organisms, scaffoldin genes encoding cohesin domains are not nearly as abundant as those encoding dockerin motifs, thus we may have simply missed capturing the cognate Clostridiaceae-derived cohesin encoding genes by chance. At 168 amino acid residues, the cohesin HMM is longer than that for a dockerin repeat (22 residues) within dbCAN. Therefore, with metasecretome library inserts being generally small in size, partial capture of cohesin sequences may not have enabled their *in silico* detection. Moreover, in case of *R. albus* strain 8, a putative cellulosome producer with many genes predicted to encode dockerin-containing enzymes for which putative cohesin domain-encoding genes have not been yet identified, it was speculated that closely related rumen bacteria may produce cognate cohesin-bearing scaffoldins that could enable appropriation of the dockerin-containing enzymes produced by *R. albus* 8 [22].

A small number of dockerin and cohesin module-containing sequences appeared to be associated with a number of bacterial families that are not known to produce cellulosomes, such as the Coriobacteriaceae, Erysipelotrichaceae and Porphyromonadaceae. It is thus uncertain whether these are from cellulosome-producing organisms. Alternatively, they may be associated with proteins that mediate roles in interactions that are not involved in cellulosomal function, but rather in proteolysis (proteases), oxidative reduction (peroxidases) or dephosphorylation (phosphatases) [64]. It has been hypothesised that in the complex ecosystems different organisms could use cohesin and dockerin modules to interact in a form of intespecies cell-cell adhesion. Alternatively, these proteins may evolve to attain different roles unrelated to cell-adhesion [64].

## Conclusions

The metasecretome phage display method combined with next-generation sequencing has the power to functionally select for, and reveal, the diversity of low-abundance surface and secreted proteins that would otherwise require large metagenomic sequencing efforts to reveal. This approach allowed the identification of a large number of cellulosomal module-containing proteins and produced a rumen microbial metasecretome display library that is currently being used to explore the roles of rumen bacterial cellulosomes and other CAZymes via standard phage display affinity selection and protein display methodologies. The novel CAZyme genes and domains identified from this study represent valuable candidates for further analysis, starting from the metasecretome library as a resource. For example, interacting pairs of cohesins and dockerins could be determined by affinity-panning of the metasecretome library using expressed cohesins as baits, whereas carbohydrate binding modules of interest could be identified by screening the metasecretome library using the complex carbohydrates as baits. Furthermore, screening of the protein repertoire displayed on the surface of metasecretome library virions for novel biocatalysts of interest [65, 66], using the reaction product-based trapping strategies or by colony-based colorimetric detection, could be used to explore the enzymatic activities that could be potentially exploited in industrial processes involving fibre degradation.

## Methods

### Rumen sampling and rumen content fractionation

A sample of whole rumen content was obtained from a fistulated Friesian dairy cow, grazing *ad libitum* on a ryegrass - clover pasture diet, supplemented with pasture silage (~10% of the recommended daily intake per animal). The sampling was conducted in May 2009 at Lye Farm, DairyNZ (Waikato, New Zealand) under the animal ethics permission number AE 11483 granted by the Ruakura Animal Ethics Committee. Between 1 and 1.5 kg of rumen contents was collected in the morning and immediately processed. A protocol for partitioning of the rumen microbial fraction tightly adherent to plant biomass (plant-adherent fraction) from liquid (planktonic) and associated (loosely attached) microbial fractions is described in detail in Additional file 4. Fractions and samples of digesta obtained from different phases of the process were snap-frozen in liquid nitrogen and kept on dry ice until long term storage at -80ºC.

### Bacterial strains, display system and growth conditions

*Escherichia coli* strain TG1 (*supE thi-1* Δ(*lac-proAB*) Δ(*mcrB-hsdSM*)*5* (r_K_^−^ m_K_^−^) [F’ *traD36 proAB lacI*^q^*Z*Δ*M15*]) was used as a host for the construction of phage display libraries, as well as for propagation of the wild-type helper phage, VCSM13 (Stratagene, USA). The *E. coli* strain K1976 (TG1 transformed with plasmid pJARA112 that expresses *gIII* under the control of phage-inducible promoter p*psp*) was used to obtain infectious stocks of the helper phage VCSM13d3, containing deletion of the complete *gIII* coding sequence [67].

Phagemid vector pDJ01 [40], designed for selective secretome display, was used for construction of the metasecretome libraries. The display cassette of pDJ01 contains the promoter p*psp*, followed by the ribosome-binding site, the start (ATG) codon, multiple cloning site and the sequence encoding the C-domain of phage protein pIII. In contrast to other display vectors, pDJ01 does not have a signal sequence. This vector also contains a chloramphenicol resistance marker (Cm^R^), plasmid (ColE1) origin of replication, and phage intergenic sequence containing f1 origin of replication and packaging signal. When helper phage VCSM13d3 is used to assemble phagemid-containing virion particles (PPs), empty pDJ01 vector only produces defective particles that are sensitive to the detergent sarcosyl [0.1% (w/v)]. Inserts that contain a signal sequence or other motifs that can mediate targeting the N-terminus of the fusion into the *E. coli* membrane or the periplasm are required for assembly of the pIII C-domain into the virion and formation of detergent-resistant virions ([40]; Figure 1).

*E. coli* cells were incubated in 2 × Yeast Extract Tryptone broth (2 × YT) at 37ºC with aeration (200 rpm). Solid medium for growth of *E. coli* transformants also contained 1.5% (w/v) bacteriological agar (Oxoid, USA) unless otherwise indicated. When required, antibiotics were added to media at the following concentrations: 25 μg ml^−1^ chloramphenicol (Cm) and 60 μg ml^−1^ ampicillin (Amp).

### Metagenomic DNA extraction from rumen microbial community plant-adherent fraction

High molecular weight metagenomic DNA from the rumen microbial plant-adherent fraction was extracted according to Stein et al. [68] with some modifications. In total, 2 g of microbial cell pellet from the plant-adherent fraction was split into five samples which were each separately embedded in 0.7 ml of 1% low-melting-temperature agarose and incubated in a syringe for 10 min on ice. Samples were extruded into 10 ml of lysis buffer [1% (w/v) sarcosyl, 0.2% (w/v) sodium-deoxycholate, 10 mM Tris-HCl (pH 8.0), 50 mM NaCl, 100 mM ethylenediaminetetraacetic acid (EDTA), lysozyme (1 mg/ml)] and incubated for 2.5 h at 37°C, followed by 17 h incubation in 40 ml ESP buffer [0.5% (w/v) sarcosyl, 20 mM EDTA and 0.013 AU protease (Qiagen, Germany)] at 55°C to inactivate nucleases present in the sample. After addition of fresh ESP buffer (20 ml) to each sample and 1 h incubation at 55°C, three washes with TE buffer [10 mM Tris-HCl (pH 8.0), 1 mM EDTA] were performed and remaining proteases were inactivated for 15 min at 70°C. To digest agarose, samples were incubated overnight at 37°C with 15 U of Agar*ACE*™ enzyme (Promega, USA). Residual insoluble oligosaccharides were removed by centrifugation and the supernatant, containing crude DNA released from the agarose, was subjected to phenol:chloroform:isoamyl alcohol extraction (25:24:1). After pooling together the five starting samples, metagenomic DNA was concentrated using a 100 kDa cut-off Vivaspin filter device (Sartorius Stedim Biotech, Germany).

### Construction of rumen metagenome phage display libraries

Two shotgun metagenome phage display libraries were constructed: a small pilot library for preliminary assessment of methodology and a large library. Both libraries were constructed from mechanically sheared metagenomic DNA isolated from the rumen plant-adherent microbial fraction and cloned into the secretome-selective phagemid pDJ01 [40] (Figure 1). Around 150 μg of high molecular weight metagenomic DNA in 55 mM Tris-HCl (pH 8.0), 15 mM MgCl_2_, 25% glycerol was sheared by nebulisation in disposable medical nebulisers by subjecting the sample to a pressure of 10 psi for 1 min, followed by size fractionation, de-salting and concentration in 100 kDa cut-off Vivaspin ultra-filtration spin columns (Sartorius Stedim Biotech, Germany). Prior to cloning, the ends of the metagenomic DNA fragments were repaired using an enzyme cocktail containing T4 DNA Polymerase (Roche, Switzerland), Klenow Enzyme (Roche, Switzerland), and OptiKinase^TM^ (Affymetrix, USA). Next, DNA was purified by phenol:chloroform:isoamyl alcohol (25:24:1) extraction followed by ethanol-precipitation and resuspension in 150 μl of 10 mM Tris-HCl (pH 8.0). Approximately 19 μg of the end-repaired metagenomic DNA inserts were ligated to 6.5 μg of the vector pDJ01, which was cut using SmaI restriction endonuclease (Roche, Switzerland) and dephosphorylated using rAPid Alkaline Phosphatase (Roche, Switzerland). Ligated DNA was extracted with phenol:chloroform, precipitated and dissolved in 75 μl sterile deionised water.

A total of 2 μg of ligated metagenomic DNA was electro-transformed into the *E. coli* TG1 electrocompetent cells to obtain the pilot shotgun library, while the rest of the ligation mixture was used in 27 separate transformation reactions to generate a large shotgun library and overcome a problem of promiscuous (fast growing) clones. The resulting 27 transformant samples were also individually processed through the whole metasecretome selection procedure and pyrosequencing sample preparation. To estimate primary shotgun library size, aliquots from each transformation were plated on Cm-containing plates. The remaining portion of each transformation mixture was mixed with 9 ml of 2 × YT broth containing chloramphenicol (2 × YT Cm_25_) and incubated for 8 h at 37°C with aeration to amplify the libraries. Amplified library aliquots were frozen at -80°C in 7% DMSO, apart from 1 ml that was used immediately for the secretome selection.

### Selection of secretome-encoding library clones

A protocol described previously with modifications was used for direct selection of the metasecretome phage display library [40]. In order for a secretome protein-encoding library to be enriched, it had to fulfil two conditions: i) to be translationally fused (i.e. in-frame) with phage protein pIII encoded by the vector; ii) to encode for a membrane-targeting signal, in order to target vector-encoded phage protein pIII (devoid of signal sequence) to the inner membrane of *E. coli*. When both of these conditions are met, the peptide fused to pIII allows display of the fusion protein on the surface of the virion and complementation of the assembly defect in the *gIII*-deletion helper phage VCSM13d3, resulting in detergent-resistant virions (phagemid particles). Selection for secretome-encoding inserts is therefore based on treatment of the library, in the form of phagemid particles, that eliminates detergent-sensitive, while preserving the detergent-resistant phagemid particles [40, 41]. A 1 ml aliquot of the overnight culture containing amplified primary library clones was used to inoculate 100 ml of 2 × YT Cm_25_ media. The exponentially growing culture (OD_600_ = 0.2) was infected with helper phage VCSM13d3 at a multiplicity of infection 50 (50 phage : 1 bacterium) for 1 h at 37°C. Infected cells were harvested by centrifugation at 2,600 × *g* for 10 min at room temperature and the resulting pellet was mixed with 40 ml of soft agar [2 × YT broth containing 0.6% (w/v) molecular biology grade agarose]. Agarose-embedded cells were poured over 16 selective plates (2 × YT Cm_25_ plates containing molecular biology grade agarose instead of bacteriological agar) and incubated overnight at 37°C [69]. Phagemid particles were extracted from each plate with 5 ml of 2 × YT, concentrated by PEG/NaCl precipitation and resuspended in 1 ml 10 mM Tris-HCl (pH 7.6).

To eliminate structurally unstable virions (lacking pIII; derived from non-secretome library clones), extracted phagemid particles were incubated in 0.1% (w/v) sarcosyl for 10 min at room temperature. The ssDNA released from defective virions was removed by incubation with DNaseI (200 U) in the presence of MgCl_2_ (5 mM) for 1 h at room temperature, followed by addition of EDTA (to final concentration of 25 mM) and heating at 75°C for 10 min to inactivate DNase. Sarcosyl-resistant recombinant virions were precipitated by PEG/NaCl and the ssDNA was extracted using E.Z.N.A.® M13 DNA Kit (Omega Bio-Tek, USA) according to manufacturer’s recommendations.

### Construction of pilot metasecretome library and sequence analysis of randomly selected metasecretome library inserts

The ssDNA isolated after the secretome selection was transformed into *E. coli* and inserts from individual transformants analysed by Sanger sequencing. In the pilot experiment, DNA from 90 randomly selected transformants were sequenced at the Massey Genome Service (Massey University, New Zealand). All inserts were sequenced using primer pspR03 (5′-TGCCTTTAGCGTCAGACTGTAGC-3′), complementary to the pIII-coding sequence of the vector to identify the insert-pIII joint and determine the frame of the insert-containing ORF relative to pIII. The sequences obtained were analysed using Vector NTI® Advance 11 Software package (Life Technologies, USA). Types of secretion signals in putative ORFs (longer than 24 amino acid residues) in frame with phage *gIII* were predicted using a range of available algorithms (SignalP 4.1 [33], TMHMM 2.0 [35], LipoP 1.0 [70], PRED-LIPO [36], SecretomeP 2.0 [71], PilFind 1.0 [72], PRED-TAT [73]) using the default settings and cut-off values.

### Next generation sequencing sample preparation

The secretome-selected ssDNA derived from the large-scale primary library through 27 separate ligations, library amplifications and selections was amplified in 27 separate PCR reactions (35 cycles starting from picogram amounts of ssDNA template) using hot-start PrimeSTAR® Max DNA Polymerase (Takara Bio, Japan). Primers PCRF2 (5′-GCCTGGTATCTTTATAGTCCTGTCGGGTTTCGCCA-3′) and PCRR2 (5′-GGCGACATTCAACGATTGAGGGAGGGAAGGT-3′) were designed to anneal to pDJ01, 361 bp upstream, and 367 bp downstream, of the library insert. Analysis of each of the 27 PCR reactions by agarose gel electrophoresis showed smears of different-sized products, and in addition several discernable bands, suggesting more prominent amplification of some clones. The band patterns were different in all 27 PCR reactions, suggesting that there was no single highly prominent amplification product. Moreover, the Sanger sequencing reactions of the two eluted bands showed multiple traces in the chromatogram, representing a mixture of products rather than a single product. The analysis of the PCR reactions by agarose gel electrophoresis also demonstrated that the amplicon corresponding to the empty vector (728 nt) could not be detected as a separate band. Empty vector was the single most abundant clone in the metagenomic library prior to selection, and the lack of its amplification using post-selection DNA as a template confirmed that the secretome selection step eliminated most of the “background” non-secretome-encoding recombinant phagemids, including the empty vector.

Amplicons generated in these 27 PCR reactions were pooled and fragmented by two shearing methods: restriction endonuclease AluI (Thermo Fisher Scientific, USA) treatment and mechanical shearing using nebulisers, under several conditions (see below), to obtain a fragment length range between 0.6 and 0.8 kb recommended for pyrosequencing. The sample was divided into portions and fragmented using five different conditions: 1 min AluI digestion; 3 h AluI digestion, 6 min nebulisation at 35 psi; 6 min nebulisation at 35 psi followed by 1 min AluI digestion, and 6 min nebulisation followed by 3 h AluI digestion. AluI digestions were performed with 5 U enzyme/μg DNA at 37°C and to stop the enzymatic reactions, AluI was inactivated by heating at 65°C for 20 min. Mechanical shearing of samples containing 10% (v/v) of glycerol was performed on ice, in a disposable nebuliser (Invitrogen, USA), by applying pressure at 35 psi for 6 min. Equal amounts (2.5 μg) of DNA, size-fractionated by all five methods, were mixed and a total of 12.5 μg DNA was submitted to pyrosequencing using 454 GS FLX Titanium platform (Roche, Switzerland) at Macrogen Inc. sequencing facility (Seoul, Korea; a half-plate in total). Sequencing template was prepared by the sequencing-service provider according to the Rapid Library Preparation Method Manual (Roche, Switzerland), except that the protocol commenced from the second, fragment end repair step.

### *In silico* analysis of NGS metasecretome dataset

Metasecretome pyrosequencing reads were trimmed with SeqClean [74] to remove sequences of pDJ01 vector and VCSM13d3 helper phage. Summary statistics for metasecretome reads are presented in Additional file 2. Metagenome sequence dataset obtained by shotgun sequencing of the total metagenomic DNA from the plant-adherent rumen microbial communities of two New Zealand cows, grazing a similar pasture-based diet to the cow used for the metasecretome library analysis, using Roche 454 GS FLX platform (one plate per cow; two plates in total) was analysed to provide a reference point for comparison to the metasecretome dataset. Both sequencing datasets were processed and automatically annotated using the JGI IMG/M system [50]. Functional categorisation and phylogenetic composition of annotated metasecretome and metagenome sequence datasets can be accessed through IMG/M system [75].

Protein coding genes predicted via the IMG/M system for the metasecretome and metagenome datasets (222,960 and 671,876 ORFs, respectively), as well as 2,547,270 predicted ORFs from the bovine switchgrass-adherent metagenome dataset [14], were subjected to annotation and assignment to families of carbohydrate-active enzymes (CAZymes) using dbCAN database release 3.0, based on the CAZy database as of March 2013 [54]. dbCAN output was parsed using the following cut-off values: alignment length > 80 amino acid residues, E-value < 1 × 10^−5^; otherwise E-value < 1 × 10^−3^. To remove duplicates and to analyse distinct ORFs, all dbCAN hits were clustered at 100% sequence identity threshold using CD-HIT algorithm [76] and clustered hits to cellulosome-associated modules were further analysed. The family level taxonomic assignment of ORFs containing cellulosome modules in the metasecretome was analysed based on the best BLASTP hit against the NCBI-NR database. For hits with a 40 bit-score threshold for cohesin and SLH module-containing ORFs, and a 35 bit-score threshold for dockerin-module containing ORFs, taxonomic family assignments of the host organism for the best BLAST hit were manually curated using recent bacterial classification proposals [56, 77–81].

### Availability of supporting data

The pilot metasecretome phage display library sequences supporting the results of this article are available in the GenBank repository and their accession numbers are included within Additional file 1. The metasecretome and metagenome sequence datasets supporting the results of this article can be accessed through the ‘quick genome search’ box available on the IMG/M main page using the corresponding IMG genome ID (3300000332 for metasecretome and 3300000524 for metagenome dataset), or in the NCBI BioProject database (accession ID PRJNA244109).

## Electronic supplementary material

Additional file 1: **Predicted membrane targeting signals and annotation of putative ORFs in the metasecretome pilot library.** (XLSX 44 KB)

Additional file 2: **Summary statistics of the rumen metasecretome pyrosequencing dataset.** (DOCX 29 KB)

Additional file 3: **Carbohydrate-active enzymes and associated modules identified in the rumen plant-adherent microbial metasecretome.** (XLSX 68 KB)

Additional file 4: **Whole rumen content fractionation.** (DOCX 28 KB)

## Abbreviations

2 × YT: 2 × Yeast extract Tryptone
Cm: Chloramphenicol
2 × YTCm25: 2 × YT broth or agar supplemented with 25 μg ml^−1^ chloramphenicol
soft agar: 2 × YT broth containing 0.6% (w/v) molecular biology grade agarose
Double-layer selective plates: 2 × YT Cm_25_ plates overlaid with Cm-free 2 × YT agar shortly before use
PEG: Polyethylene glycol
ORF: Open reading frame
ssDNA: Single-stranded DNA
NGS: Next-generation sequencing
HMM: Hidden Markov Model.
