# Supplementary material for: Metasecretome-selective phage display approach for mining the functional potential of a rumen microbial community
Source: BMC Genomics. 2014 May 12;15(1):356. doi: 10.1186/1471-2164-15-356 (PMC4035507; doi:10.1186/1471-2164-15-356)
Supplement: Supplementary file 2 — Additional file 2: Summary statistics of the rumen metasecretome pyrosequencing dataset. (DOCX 29 KB) [file 12864_2013_6070_MOESM2_ESM.docx]

**Additional file 2. Summary statistics of the rumen metasecretome pyrosequencing dataset.**

|  | **Raw reads^a^** | **Reads after processing with SeqClean^b^** | **Reads after processing *via* IMG/M pipeline^c^** |
| --- | --- | --- | --- |
| Number of reads | 691,206 | 492,198 | 153,002 |
| Average read length (bp) | 548 | 322 | 362 |
| Total sequence information (Mb) | 379 | 158 | 55 |

Number of reads, average read length (bp) and total sequence information (Mb) is represented for: a) Raw pyrosequencing reads, obtained by shotgun sequencing of metasecretome-enriched DNA from rumen adherent microbial fraction using Roche 454 GS-FLX Titanium platform; b) Reads after trimming phagemid vector and helper phage sequences and filtering short reads (<100 bp) using SeqClean; c) Reads after processing (including trimming, low complexity filtering and dereplication) *via* IMG/M pipeline.
