# Supplementary material for: Metasecretome-selective phage display approach for mining the functional potential of a rumen microbial community
Source: BMC Genomics. 2014 May 12;15(1):356. doi: 10.1186/1471-2164-15-356 (PMC4035507; doi:10.1186/1471-2164-15-356)
Supplement: Supplementary file 4 — Additional file 4: Whole rumen content fractionation. (DOCX 28 KB) [file 12864_2013_6070_MOESM4_ESM.docx]

**Additional file 4.** **Whole rumen content fractionation.**

Total rumen digesta was squeezed through double-layered cheesecloth and liquid microbial fraction was obtained from released fluid by centrifugation at room temperature, first at 350 x g for 15 min, followed by two centrifugation steps at 10,000 x g for 10 min. In order to detach loosely associated microbial fraction, squeezed digesta was resuspended in RM02 buffer [0.15% (w/v) potassium chloride, 0.14% (w/v) potassium di-hydrogen phosphate and 0.06% (w/v) ammonium sulphate] at room temperature and squeezed through cheesecloth. Associated fraction was obtained from the first wash by centrifugation at 350 x g for 15 min at room temperature, followed by two centrifugation steps at 10,000 x g for 10 min.

Plant-adherent rumen microbes were chemically detached from squeezed digesta that was washed four times in RM02 buffer prior to 2.5 hour-incubation in 200 ml dissociation buffer containing 0.1% sodium pyrophosphate and 0.5% Tween 20
(pH 6.8) on ice, followed by 30 min incubation at 4ºC with shaking at 290 rpm. The fluid obtained after squeezing material through cheesecloth was centrifuged at 350 x g for 15 min at room temperature to remove small plant debris, followed by centrifugation at 10,000 x g for 10 min to obtain microbial pellet. Bacterial pellet was washed once in RM02 buffer, centrifuged at 10,000 x g for 10 minutes and snap frozen in liquid nitrogen. Samples were transported on dry ice and stored at -85°C until DNA extraction.
